# Identification of Phthalates in Medications and Dietary Supplement Formulations in the United States and Canada

**DOI:** 10.1289/ehp.1103998

**Published:** 2011-12-15

**Authors:** Katherine E. Kelley, Sonia Hernández-Díaz, Erica L. Chaplin, Russ Hauser, Allen A. Mitchell

**Affiliations:** 1 Slone Epidemiology Center at Boston University, Boston, Massachusetts, USA; 2 Department of Epidemiology, Harvard School of Public Health, Boston, Massachusetts, USA; 3 Occupational and Environmental Medicine and Epidemiology Program, Department of Environmental Health, Harvard School of Public Health, Boston, Massachusetts, USA

**Keywords:** coating, dietary supplements, excipients, medications, phthalates

## Abstract

Background: In animal studies, some *ortho*-phthalates, including di(2-ethylhexyl) phthalate (DEHP) and di-*n*-butyl phthalate (DBP), have been shown to be reproductive and developmental toxicants. Human studies show widespread population exposure to background levels of phthalates. Limited evidence suggests that particularly high exposure levels may result from orally ingested medicinal products containing phthalates as excipients (inactive ingredients).

Objective: In this study we aimed to identify and describe the scope of prescription (RX) and nonprescription (over-the-counter; OTC) medicinal products and dietary supplements marketed in the United States and Canada since 1995 that include phthalates as excipients.

Methods: We used lists of modified-release drug products to identify potential drug products. Inclusion of phthalates was verified using available electronic databases, print references, published package inserts, product packages, and direct communication from manufacturers. Additional products were identified using Internet searches utilizing keywords for phthalates.

Results: Based on labeling information, 6 RX drug products included DBP as an excipient, and 45 specified the use of diethyl phthalate (DEP). Phthalate polymers with no known toxicity—hypromellose phthalate (HMP), cellulose acetate phthalate (CAP), and polyvinyl acetate phthalate (PVAP)—were included in 75 RX products. Three OTC drug and dietary supplement products listed DBP, 64 listed DEP, and > 90 indicated inclusion of polymers.

Conclusions: Numerous RX and OTC drug products and supplements from a wide range of therapeutic categories may use DBP or DEP as excipients in oral dosage forms. The potential effects of human exposure to these phthalates through medications are unknown and warrant further investigation.

Some *ortho*-phthalates, including di(2-ethylhexyl) phthalate (DEHP) and di-*n*-butyl phthalate (DBP), have been identified as reproductive and developmental toxicants in laboratory animals (Foster 2005; Howdeshell et al. 2008). Similar effects have not been reported in animals exposed to diethyl phthalate (DEP); however, limited human studies have suggested a possible association between DEP exposure and male reproductive health outcomes (Swan 2008). Studies have shown widespread population exposure to background levels of phthalates (Silva et al. 2004). Of concern, particularly high exposure levels may result from orally ingested medicinal products containing phthalates as inactive ingredients. For example, increased urinary concentrations of phthalate metabolites were first reported for an individual taking a mesalamine product (Asacol; Warner Chilcott Pharmaceuticals Inc., Mason, OH) (Hauser et al. 2004) and later confirmed for individuals taking mesalamine and other drug products (Hernández-Díaz et al. 2009). High urinary concentrations of phthalate metabolites have also been identified in patients with cystic fibrosis who used pancreatic enzyme products (Keller et al. 2009). This has led to increased interest in identifying additional drug products that contain phthalates as inactive ingredients.

Inactive ingredients (excipients) are defined as “any component other than the active ingredient in a drug product” (FDA 2011). These various compounds may be used in drug formulations for a number of functions related to the preparation and stability of the physical dosage form or the delivery of active ingredients (Rowe et al. 2009). Excipients used in specialized drug product formulations may permit *a*) localized availability of active medications in various sections of the gastrointestinal (GI) tract, *b*) controlled release of a medication over time, or *c*) through the skin. Phthalates, in combination with various polymers, may be used as plasticizers and film coating agents in orally ingested solid pharmaceutical dosage forms and in numerous types of modified-release drug delivery systems such as enteric-coated and delayed-release tablets, pelletized delayed-release capsules, enteric-coated capsules, and controlled-release transdermal films (Allen et al. 2005).

Several different phthalates are currently used as excipients in approved pharmaceutical formulations: diethyl phthalate (DEP), dibutyl phthalate (DBP), dimethyl phthalate (DMP), dioctylphthalate (DOP), hypromellose phthalate (HMP), cellulose acetate phthalate (CAP), polyvinyl acetate phthalate (PVAP), and polyethylene terphthalate (PET) [Food and Drug Administration (FDA) 2010b]. These compounds are not themselves technically “approved” for general use as excipients; rather, they are permitted to be included in their corresponding approved drug products, with maximum levels designated for each type of dosage form and route of administration (Osterberg and See 2003; Steinberg and Silverstein 2003). The maximum amount of DBP used in specific FDA-approved drug products ranges from 1.70 mg for a delayed-action, enteric-coated tablet formulation to 11.18 mg for an extended-release capsule product. DEP has been approved at amounts ranging from 0.5 mg for an uncoated, chewable tablet to 16.8 mg for a delayed-action, enteric-coated capsule (FDA 2010b).

Nonprescription pharmaceutical (over-the-counter; OTC) and dietary supplement products may contain excipients found in approved prescription (RX) products; however, their composition is not always reviewed prior to marketing. OTC drug products marketed under monograph guidelines do not require premarket review of their ingredients, but contents are reviewed for products approved under new drug applications (NDAs) and abbreviated NDAs (ANDAs) (FDA 2010a). Dietary supplements are regulated as food products under the Dietary Supplement Health and Education Act of 1994 (DSHEA 1994), and their formulations do not require approval prior to marketing unless they contain a new dietary ingredient (FDA 2009).

Manufacturers are required to disclose a listing of inactive ingredients in drug product labeling, except when they are used in patented delivery mechanisms where the composition is considered to be a confidential trade secret (U.S. Pharmacopeial Convention 2009). Although the FDA might have information on the type and amount of phthalates used in specific approved drug products, they cannot make the information public because of its proprietary nature.

In this study we sought to identify and describe the scope of medicinal pharmaceutical products and dietary supplements marketed in the United States and Canada since 1995 that could be confirmed to contain phthalates as excipients based on available product information supplied by regulators, manufacturers, labelers, distributors, and marketers of these products.

## Methods

A search of the published literature on the use of phthalates in orally ingested medications suggested that phthalates are frequently included as inactive ingredients in modified-release drug formulations (i.e., controlled-release, delayed-release, or targeted-release systems), as well as in formulations for film coatings. Drug products using these formulations rely on the integrity of their coating systems for proper delivery of their active ingredients and therefore should not be crushed or chewed. Lists of drug products that are not recommended to be crushed or chewed have been published for reference by health care professionals to ensure their proper administration (Mitchell 2011; Thomson PDR 2006). We used these lists, which included approximately 450 product names, to complete an initial screen of drug product labeling for the inclusion of phthalates as excipients.

All inactive ingredients for RX medications on the “do not crush list” were first reviewed using a combination of print and electronic resources including the *Physicians’ Desk Reference* (Thomson Healthcare 1995–2010a), FDA Approved Drug Products database (Drugs@FDA; FDA 2011b), DailyMed [National Library of Medicine (NLM) 2011a], and LabelDataPlus (Reed Technology and Information Services Inc. 2011). Canadian products with comparable active ingredients and formulations were researched by using any available monographs from the Health Canada Drug Product Database (Health Canada 2011a) and the *CPS: Compendium of Pharmaceuticals and Specialties* (Canadian Pharmaceutical Association 1995–2006). Names of brand and generic drug products marketed since 1995 appearing on the lists were searched within each available electronic or print reference, and all potential modified-release formulations were selected from the results. Once a label was located, we searched the document for all inactive ingredients. If multiple labels were available within a database, we investigated all potential inactive ingredient formulation changes by reviewing the older labels. If an older formulation of the product contained phthalates but the newer formulations did not, we searched all available labels by revision date to determine when the formulation changed. If none of the available compendia provided sufficient label information for a product, we searched the manufacturer or distributor website. If no label was found, we contacted the manufacturer and requested a copy of the package insert.

We performed further Internet searches using terms containing “phthalate” and “medication,” or suspected medication names and drug classes (e.g., bisacodyl, enzyme), to locate additional products and product categories. Patents for proprietary drug delivery systems were reviewed for the use of phthalate plasticizers in specific drug products or groups of products.

Several OTC medications and dietary supplement products were listed on the “do not crush” list; however, most available references including the *Physicians’ Desk Reference for Nonprescription Drugs, Dietary Supplements, and Herbs* (Thomson Healthcare 1995–2010b), Drugs@FDA, LabelDataPlus, DailyMed, and Dietary Supplements Labels Database (NLM 2011b) either did not include these products or did not provide complete, searchable label information on their inactive ingredients. For these products, we obtained detailed information on inactive ingredients from manufacturer and distributor web sites, or if possible, directly from product package labeling retrieved from store shelves. Canadian dietary supplement products were searched using the Health Canada Licensed Natural Health Products Database (LNHPD; Health Canada 2011b), which currently contains information on approximately 16,000 licensed dietary supplements available in solid oral dosage forms.

## Results

*Use in prescription drugs.* Various phthalates were identified in RX medications available in the United States and Canada across a wide variety of therapeutic classes, including analgesic, antibiotic, GI, cardiovascular, and antiepileptic drug products. As expected, we found that phthalates were most commonly used in modified-release RX drug formulations that provided timed-release and targeted-release of active ingredients. Of the RX products that were identified, 75 contained the phthalate polymers HMP, CAP, and PVAP (data not shown), and 50 contained the *ortho*-phthalates DEP and/or DBP (6 contained DBP, and 1 contained both DEP and DBP) (Table 1).

**Table 1:** Prescription drug products marketed in the United States and Canada listing *ortho*-phthalates as inactive ingredients in product labeling, 1995–2011.

Generic name	Trade name*a*	Labeler/manufacturer	Phthalate	Drug class
Acebutolol		Rhotral 400 mg tablets (Canada)(discontinued 2006)		Rhodiapharm (Canada)		DEP		Cardiovascular/beta blocker
Carbamazepine		Carbamazepine 200 mg tablet		Teva Pharmaceuticals USA Inc.		DEP		Antiepileptic
		Taro-Carbamazepine CR (Canada)		Taro Pharmaceuticals Inc. (Canada)		DEP		
Cyclobenzaprine		Amrix		Cephalon Inc.		DEP		Muscle relaxant
		Cyclobenzaprine HCl Extended Release Capsules		CIMA Labs Inc./Eurand		DEP		
Didanosine		Didanosine, Enteric Coated		Aurobindo Pharma Ltd.		DEP		Antiretroviral
		Didanosine, Enteric Coated		Mylan Pharmaceuticals Inc.		DEP		
		Videx EC (United States and Canada)		Bristol Myers Squibb Co./Bristol Myers Squibb Canada		DEP		
Diltiazem		Cardizem CD 360 mg Capsule		BTA Pharmaceuticals Inc.		DEP		Cardiovascular/CCB
		Dilt-CD		Apotex Corp.		DBP		
		Diltiazem Extended Release (Twice-A-day)		Mylan Pharmaceuticals Inc.		DEP		
Divalproex		Divalproex sodium tablet, delayed release		Aurobindo Pharma Limited		DEP		Antiepileptic
		Divalproex sodium tablet, delayed release		Greenstone LLC/Aurobindo Pharma		DEP		
Erythromycin		ERYC (Canada)		Pfizer Canada Inc.		CAP, DEP		Antibiotic
Ethopropazine		Parsitan (Canada)		Rhone-Poulenc Rorer/Erfa Canada Inc.		DEP		Antiparkinsons
Galantamine		Razadyne ER		Ortho-McNeil Neurologics		DEP		Antiparkinsons
		Galantamine HBr Extended Release Capsules		Global Pharmaceuticals/Impax Laboratories Inc.		DEP		
		Reminyl ER (Canada)		Janssen-Ortho Inc.		DEP		
Isosorbide		Isosorbide Mononitrate, tablet extended release		Torrent Pharmaceuticals Ltd.		DEP		Cardiovascular/vasodilator
Ketoprofen		Nu-Ketoprofen E (Canada)		Nu-Pharm (Canada)		CAP, DEP		NSAID
		Orudis SR (Canada) (discontinued 2005)		Aventis Pharma (Canada)		CAP, DEP		
Mesalamine		Asacol (United States and Canada)		Warner Chilcott US LLC/Warner Chilcott Canada Co.		DBP		GI/anti-inflammatory
		Asacol HD		Warner Chilcott US LLC.		DBP		
		Asacol 800 (Canada)		Warner Chilcott Canada Co.		DBP		
		Salofalk 500 mg Enteric Coated (Canada)		Axacan Pharma Inc.		DEP		
Morphine sulfate		Kadian		Actavis Kadian; Alpharma Branded Products		DEP		Analgesic
		Morphine Sulfate Extended Release Capsules		Actavis Elizabeth LLC		DEP		
Morphine sulfate/naltrexone		Embeda		King Pharmaceuticals/Actavis		DEP		Analgesic
Pancrelipase		Cotazym ECS (OTC in Canada)		Organon Canada Ltd./Schering-Plough Canada Inc.		CAP, DEP		GI/enzyme
		Creon Minimicrospheres		Solvay Pharmaceuticals Inc.		DBP, HMP		
		Lipram		Eurand America		DEP, HMP		
		Pancrease		McNeil		CAP, DEP		
		Pancrecarb		Digestive Care Inc.		CAP, DEP		
Potassium chloride		Potassium Chloride Extended Release Tablets, USP		American Health Packaging/Watson/Eurand		DEP		Potassium supplement
		Riva-K 20 SR (Canada)		Laboratories Riva Inc. (Canada)		DEP		
Prochlorperazine		Stemetil tablets (Canada) (discontinued 2005)		Rhone-Poulenc Rorer/Aventis Pharma		DEP		Antiemetic
Propranolol		InnoPran XL		GlaxoSmithKline		DEP, HMP		Cardiovascular/beta blocker
Ranitidine		Ranitidine Tablets, USP 150 mg		Wockhardt USA, LLC		DEP		GI/antiulcer-H2 Blocker
		Ranitidine Tablets, USP 300 mg		Wockhardt USA, LLC		DEP		
Sulfasalazine		Sulfasalazine Delayed Release Tablets, USP		Vintage Pharmaceuticals		CAP, DEP		GI/anti-inflammatory
		Sulfazine EC		Qualitest/Vintage Pharmaceuticals-Charlotte		CAP, DEP		
Theophylline		Theo-Dur Extended Release Tablets		Key Pharmaceuticals		CAP, DEP		Respiratory/bronchodilator
		Uni-Dur Extended Release Tablets		Key Pharmaceuticals		CAP, DEP		
Thioproperazine mesylate		Majeptil (Canada)		Rhone-Poulenc Rorer/Erfa Canada Inc.		DEP		Antipsychotic
Trimipramine		Rhotrimine Tablets (Canada)(discontinued 2006)		Rhodiapharm (Canada)		DEP		Antidepressant
Typhoid vaccine, oral		Vivotif (Canada)		Crucell Switzerland Ltd.		DBP, DEP, HMP		Vaccine
Valproic acid		ratio-Valproic 500 mg Capsules (Canada)		ratiopharm (Canada)		CAP, DEP		Antiepileptic
		phl-Valproic Acid 500 mg Capsules (Canada)		Pharmel Inc.		CAP, DEP		
Verapamil		Verapamil HCL Extended Release Capsules		Mylan Pharmaceuticals Inc.		DEP		Cardiovascular/CCB
		Verapamil HCL PM Extended Release Capsules		Mylan Pharmaceuticals Inc.		DEP		
Abbreviations: CCB, calcium channel blocker; NSAID, nonsteroidal anti-inflammatory drug. **a**Applies to all strengths and dosage forms unless otherwise noted.								

*Use in OTC drugs and dietary supplements.* Enteric coatings, film coatings, and specialized capsule formulations utilizing phthalates were noted for a number of different product categories and purposes in OTC drugs and supplements. These functions included prevention of premature degradation of substances such as enzymes, acid reducers, and probiotics by stomach acid; reduce stomach upset from iron and laxatives; minimize the aftertaste from fish oil and garlic; and produce soft gelatin capsules (i.e., “softgels”) that may be easier to swallow or contain solubilized drugs and oils (e.g., ibuprofen, fish oil).

Thirty-eight of the OTC drug products (Table 2) and 24 of the dietary supplement products (Table 3) located listed DEP, and 93 included phthalate polymers (data not shown). Although there were only 3 ANDAs for OTC ranitidine detailing the use of DEP, they were linked to label information for an additional 31 private label antacid products. Only 1 OTC drug available both in the United States and Canada [Dulcolax Laxative Tablets (Boehringer Ingelheim Pharmaceuticals, Ridgefield, CT, USA; and Boehringer Ingelheim (Canada) Ltd./Ltée, Burlington, Ontario, Canada] and 2 older formulations of dietary supplement products listed DBP [Venastat Leg Vein Health (Boehringer Ingelheim Pharmaceuticals) and Wobenzym N (Mucos Pharma, Průhonice, Czech Republic)].

**Table 2:** Nonprescription drugs products marketed in the United States and Canada listing *ortho*-phthalates as inactive ingredients in product labeling, 1995–2011.

Generic name	Trade name*a*	Labeler/manufacturer	Phthalate	Drug class
Brompheniramine		Dimetane Allergy Extentabs		Whitehall-Robins		CAP, DEP		Antihistamine
Aspirin-delayed release		Entrophen ECT 975 mg (Canada)		Pendopharm, Division of/de Pharmascience Inc (Canada)		CAP, DEP		Analgesic/antipyretic
		Enteric Coated A.S.A. (Canada)		Pendopharm, Pharmascience (Canada)		CAP, DEP, PVAP		
Bisacodyl		Dulcolax Bisacodyl Laxative Tablets		Boehringer Ingelheim		DBP		GI/laxative
		Dulcolax ECT 5 mg (Canada)		Boehringer Ingelheim Canada Ltd.		DBP		
Omeprazole		Omesec Delayed Release Capsules		Corporation Infarmasa		DEP, HMP		GI/antiulcer-PPI
Ranitidine		Ranitidine 75 mg (Wockhardt)*b *		Wockhardt USA LLC		DEP		GI/antiulcer-H2 blocker
		Ranitidine 150 mg (Wockhardt)*c *		Wockhardt USA LLC		DEP		
		Ranitidine 75 mg (Wockhardt)*d *		Wockhardt USA LLC		DEP		
		Berkley & Jensen Acid Reducer 150 mg*c *		BJWC		DEP		
		Care One Acid Reducer 75 mg*b *		American Sales Company		DEP		
		CVS Maximum Strength Acid Reducer*c *		CVS Pharmacy		DEP		
		DG Health Acid Reducer 75 mg*b *		Dolgencorp Inc.		DEP		
		DG Health Acid Reducer 150 mg*c *		Dolgencorp Inc.		DEP		
		Equaline Acid Reducer Ranitidine 150 mg*c *		Supervalu Inc.		DEP		
		Equaline Heartburn Relief 75 mg*b *		Supervalu Inc.		DEP		
		Formucare Ranitidine 75*b *		Access Business Group International LLC		DEP		
		Good Neighbor Acid Reducer 150 mg*c *		Amerisource Bergen		DEP		
		Good Neighbor Acid Reducer 75 mg*b *		Amerisource Bergen		DEP		
		Goodsense Acid Reducer 150 mg*c *		L Perrigo Company		DEP		
		Goodsense Acid Reducer 75 mg*b *		L Perrigo Company		DEP		
		Kirkland Signature Acid Reducer*c *		Costco Wholesale Corporation/Perrigo		DEP		
		Kroger Heartburn Relief 75 mg*b *		Kroger Company		DEP		
		Leader Acid Control 75 mg*b *		Cardinal Health		DEP		
		Leader Acid Control 150 mg*c *		Cardinal Health		DEP		
		Major Ranitidine 150 Maximum Strength*c *		Major Pharmaceuticals		DEP		
		Major Ranitidine 75*b *		Major Pharmaceuticals		DEP		
		Medicine Shoppe Acid Reducer 75 mg*b *		Medicine Shoppe International		DEP		
		Meijer Acid Reducer 75 mg*b *		Meijer Distribution Co		DEP		
		Publix Acid Reducer 75 mg*b *		Publix Super Markets Inc.		DEP		
		Safeway Acid Reducer 150 mg*c *		Safeway		DEP		
		Safeway Acid Reducer 75 mg*b *		Safeway		DEP		
		Sunmark Acid Reducer 150 mg*c *		McKesson		DEP		
		Sunmark Acid Reducer 75 mg*b *		McKesson		DEP		
		TopCare Heartburn Relief 75 mg*b *		Topco Associates LLC		DEP		
		TopCare Heartburn Relief 150*c *		Topco Associates LLC		DEP		
		UP & UP Acid reducer 75 mg*b *		Target		DEP		
		Wal-Zan 75 Acid Reducer*b *		Walgreens/Perrigo		DEP		
		Wal-Zan 150 Acid Reducer*c *		Walgreens/Perrigo		DEP		
		Western Family Foods Acid Relief 75 mg*b *		Western Family Foods Inc.		DEP		
Abbreviations: NSAID, nonsteroidal anti-inflammatory drug; PPI, proton pump inhibitor. **a**Applies to all strengths and dosage forms unless otherwise noted. **b**ANDA 076760; manufacturer, Wockhardt, Mumbai, India. **c**ANDA 078653; manufacturer, Wockhardt, Mumbai, India. **d**ANDA 078884; manufacturer, Wockhardt, Mumbai, India.								

**Table 3:** Dietary supplements marketed in the United States and Canada listing *ortho*-phthalates as inactive ingredients in product labeling, 1995–2011.

Generic name	Trade name	Manufacturer/distributor	Phthalate	Supplement type
Fish oil		Omega 3+ Enteric Coated Fish Oil		Health Thru Nutrition/Epic4Health		DEP, HMP		Nutritional
		Phyto-Therapy Omega 3 MLC Fish Oil, Enteric coated		Phytotherapy USA		DEP, HMP		
Garlic		Walgreens Finest Natural Garlic, tablets		Walgreens		DEP		Herbal
		Wholehealth GarlicHealth, Odorless Garlic		Wholehealth		CAP, DEP		
		Nikken Garlic (Bio-Directed Nutri-Technology)		Nikken		CAP, DEP		
		Planetary Herbals GarliChol		Planetary Herbals		CAP, DEP		
		Top Care Garlic Tablet		Perrigo		DEP		
		Ail Garlic (Canada)		Lyo-San Inc. (Lachute, Quebec, Canada)		CAP, DEP		
Magnesium		Slow-Mag Magnesium Chloride with Calcium		Purdue Products LP		DEP		Mineral
Vitamin B		Tri-Phos-B		Montiff Inc.		CAP, DEP		Vitamin
Vitamin C		Viva Vitamin C 500 mg tablets (Canada)		Viva Pharmaceutical Inc. (Canada)		DEP		Vitamin
		Viva Vitamin C 1,000 mg tablets (Canada)		Viva Pharmaceutical Inc. (Canada)		DEP		
Probiotics		Advanced Naturals Ultimate FloraMAX (Canada)		Renew Life Canada Inc.		CAP, DEP		Probiotic
		Acidophilus Complex with FOS (Canada)		WN Pharmaceuticals Ltd. (Canada)		CAP, DEP		
		Dophilus Select Plus (Canada)		Les Importations Biochala Inc. (Canada)		CAP, DEP		
		Swiss Natural 5 Strain Dophilus Capsules (Canada)		Swiss Herbal Remedies Ltd./TheraPro (Canada)		CAP, DEP		
		Primadophilus Reuteri (Canada)		Nature’s Way of Canada Ltd.		CAP, DEP		
		Bifidus (Canada)		Lyo-San Inc. (Canada)		CAP, DEP		
		Probioflor (Canada)		Lyo-San Inc. (Canada)		CAP, DEP		
		Lacto-B (Canada)		Lyo-San Inc. (Canada)		CAP, DEP		
		Probiolomgum (Canada)		Lyo-San Inc. (Canada)		CAP, DEP		
		Protec (Canada)		Natural Factors Nutritional Products Limited (Canada)		CAP, DEP		
Enzymes		Wobenzym N		Mucos Pharma		DBP		Enzyme
Misc combinations		Venastat Leg Vein Health (until 2005)		Pharmaton/Boehringer Ingelheim		DBP		Natural product
		Bio ATP		Bio Active Nutritional		CAP, DEP		Vitamin/Herbal
		Shenyang Hongyao Film Coated tablet (Canada)		Wing Quon Enterprises Ltd.		DEP, HMP		Herbal
Misc, miscellaneous.								

*Canada.* Several RX and OTC medications approved for use in both the United States and Canada contained the same inactive ingredients in the product labeling (e.g., Asacol, Dulcolax) (Tables 1 and 2); however there were some differences. Of particular note, the U.S. product labeling for Vivotif (Typhoid Vaccine Live Oral Ty21a) (Berna Biotech Ltd. 2006) did not detail the use of any phthalates, whereas the monograph for the Canadian product (Crucell Switzerland LTD 2010) specified use of “3–8 mg” of DBP and DEP and “27–33 mg” of HMP in the lacquer coating of each capsule. Several U.S. products were not available in Canada (e.g., Cardizem CD 360 mg, Dilt-CD), and some Canadian products were not distributed in the United States (e.g., Rhotral, Parsitan). In the LNHPD (Health Canada 2011b), 49 licensed supplement products were linked to phthalates as nonmedicinal ingredients, including 14 noted as containing DEP (Table 3).

*Changes over time.* A handful of products changed the use of specific excipients over time either by removing specific phthalates or adding phthalates to newly approved formulations. Solvay Pharmaceuticals removed DBP from its new FDA-approved formulations of pancrelipase capsules in April 2009. The older formulation, Creon Minimicrospheres, (Solvay Pharmaceuticals Inc. 2006), which contained DBP and HMP, was replaced with Creon Delayed Release Capsules (Abbott Laboratories 2011). New formulations for two strengths of OTC and RX ranitidine tablets from one manufacturer (Wockhardt USA LLC, Parsippany, NJ) that were approved in 2007, 2008, and 2009 (Wockhardt USA LLC 2007, 2008, 2009) used DEP as a film coating agent, whereas labels for > 30 earlier approved ranitidine products did not list any phthalates.

In May 2010, Warner Chilcott modified the labeling for Asacol and Asacol HD under the sections “Precautions” and “Use in Specific Populations.” The new labels provided cautions on use during pregnancy based on animal studies of DBP exposure and also added the estimated human daily intake of DBP from the maximum recommended daily dose of each product, “about 21 mg” and “about 48 mg” for Asacol and Asacol HD, respectively (Warner Chilcott Pharmaceuticals Inc. 2010a, 2010b)

*Use in specific formulations.* Inclusion of phthalates—as well as the specific type of phthalate—in formulations for a particular drug was manufacturer specific, and on occasion dosage-form and strength specific. For example, the calcium channel blockers verapamil and diltiazem were available from 18 manufacturers in a number of strengths and time-release tablet and capsule formulations that contained different excipients. Although we found that DEP was included in two verapamil extended-release capsule products, both from one manufacturer (Mylan Pharmaceuticals Inc. 2010a, 2010b), other tablet and capsule formulations did not contain DEP. Among diltiazem products, only one of five strengths of Cardizem CD capsules (BTA Pharmaceuticals Inc. 2010) listed DEP, whereas manufacturers of other specific diltiazem capsule products utilized phthalates across all strengths: DBP in Dilt-CD (Apotex Inc. 2011), DEP in Diltiazem Extended-Release, Twice-A-Day (Mylan Pharmaceuticals Inc. 2006), and HMP in Diltia XT (Watson Pharma Inc. 2008).

*Other sources of information.* Detailed Internet searches led to the identification of two phthalate-containing products in patented release mechanisms, despite the fact that phthalates were not listed in the corresponding product labels. In a lawsuit brought against several manufacturers attempting to introduce new generic omeprazole products into the market in 2001, the manufacturer of Prilosec Delayed Release Capsules (AstraZeneca Pharmaceuticals LP, Wilmington, DE) argued that the new formulations infringed on certain sections of their patent involving the use of phthalates, including DEP and HMP, as coating agents (Astra Aktiebolag et al. *v*. Andrx Pharmaceuticals Inc., et al., 2002). Communication with the manufacturer (AstraZeneca, personal communication) confirmed the use of HMP in the enteric coating layer of the pelletized drug found within the capsules. A similar patent infringement case (Shire LLC *v*. Nostrum Pharmaceuticals Inc. 2008) detailed the use of HMP in patents for carbamazepine extended release capsules (Carbatrol; Shire US Inc., Wayne, PA).

## Discussion

In this study, we identified a number of different phthalates as excipients in a variety of pharmaceutical products and dietary supplements sold in the United States and Canada. Although most of these products included phthalate polymers that are considered to be of low or no known toxicity, label information for several product formulations contained the phthalate diester DBP, which has been shown to cause adverse effects in experimental animal studies.

Although we attempted to review as many product listings as possible, this list cannot be considered comprehensive. Information on a number of products was unavailable due to product discontinuation and changes in manufacturers. Products may have changed their formulation over time, and the information available at the time of this review may not reflect the most recent version of the product formulation. In addition, some product information was incomplete (all inactive ingredients were not listed), which required that we seek information directly from the manufacturer. Although the FDA might have a more complete listing of products, including the type and amount of phthalate in specific drug products, this information is considered proprietary and cannot be accessed. Given the thousands of orally ingested products on the market (RX, OTC, and dietary supplements), it is difficult to quantify the prevalence of phthalate-containing products. However, it is informative to identify specific drug products that have included phthalates in their formulations.

It is also important to note the limitations and difficulties that were associated with some of the references used to compile the information for this study. Information on the specific excipients used in these products was generally available; however, it was not centrally located, comprehensive, or easily queried.

The FDA Approved Drug Products Database (Drugs@FDA) did not contain labels for all products, especially generics, and only some entries contained copies of approval history documents that detail product formulations. Several additional labels were available on LabelDataPlus, DailyMed, or manufacturer web sites, demonstrating that electronic versions of the labels were available but were not linked to the FDA database. For example, detailed label information for prescription and OTC ranitidine tablets from Wockhardt was available on DailyMed and LabelDataPlus but not on Drugs@FDA. The available OTC labels linked these approved products to labeling for several private label distributors (e.g., mass merchandise, grocery and drug store chains); however, these labelers and distributors often change the source for their private-label products over time. Neither Drugs@FDA nor Health Canada’s Drug Product Database permitted a systematic search of excipients in RX and OTC drug products. LabelDataPlus permitted queries for inactive ingredients; however, it was not comprehensive.

The *Physicians’ Desk Reference* (Thomson Healthcare 1995–2010a) contained some additional product labels, especially older versions of labels and discontinued products (e.g., Theo-Dur; Key Pharmaceuticals Inc., Kenilworth, NJ); however, available information was limited to manufacturers who elected to include their products in the reference.

We found several NDAs and ANDAs containing information on excipients using Drugs@FDA. However, because of the proprietary nature of the formulations, in most instances information concerning the type and amount of inactive ingredients used were redacted from the available documents. In LabelDataPlus, the strength of each inactive ingredient was provided for one omeprazole product—Omesec Delayed Release Capsules—listed as containing 0.144 mg DEP (Corporacion Informasa 2010); however, this was the only drug product for which we found this level of information to be available.

For a number of dietary supplement products, the available information on inactive ingredients was incomplete or unclear. Available label information sometimes described products as having an “enteric coating” without a specific listing of the components, making it impossible to determine whether any phthalates were present. Some fish oil capsules labels stated that they “may also contain” a variety of excipients, including HMP. Canada’s LNHPD (Health Canada 2011b) and the NLM Dietary Supplements Labels Database permitted searches by inactive ingredients for licensed health products and supplements, but these databases are not comprehensive directories of marketed products.

In order to create a comprehensive listing of phthalate-containing products, individual manufacturers would have to provide information on their use of phthalates in specific product formulations. Such a list (similar to those available for other excipients such as gelatin or gluten) would not only facilitate critical research on the risks and safety of phthalate-containing medications but also would allow prescribers and consumers to select alternative products that are phthalate free. Estimates of exposure from these products can vary according to manufacturer-specific formulation characteristics, including the dosage form, dose, date of use, and country of manufacture. Researchers, consumers, and health care providers may need to contact manufacturers directly to confirm the presence of phthalates and the amounts used in a particular drug product.

## Conclusion

Using a combination of resources, we were able to identify > 100 drug and dietary supplement products that indicated the use of *ortho-*phthalates as excipients, including labels of 50 RX, 40 OTC, and 26 dietary supplement product formulations that listed DEP and/or DBP (9 contained DBP, and 1 contained both DEP and DBP). The potential effects of human exposure to these phthalates through medications are unknown and warrant further investigation. The present findings should assist researchers in conducting the necessary studies of risk and safety of phthalates in human populations, but such efforts are limited by the lack of centralized, comprehensive, and publically available information on the presence of phthalates in the full range of RX, OTC, and dietary supplement products. Future research should pay particular attention to the amount of phthalate, specifically DBP, used in each dosage form so that estimates of exposure from medications and supplements can be quantified.
